# Injury prevention for older adults: A dataset of safety concern narratives from online reviews of mobility-related products

**DOI:** 10.1016/j.dib.2022.108044

**Published:** 2022-03-15

**Authors:** Felipe Restrepo, Namrata Mali, Laura P. Sands, Alan Abrahams, David M Goldberg, Janay White, Laura Prieto, Peter Ractham, Richard Gruss, Nohel Zaman, Johnathon P. Ehsani

**Affiliations:** a Department of Industrial and Systems Engineering, Virginia Tech, 250 Durham Hall (MC 0118) 1145 Perry Street, Blacksburg, VA 24061, USA; b Department of Computer Science, Virginia Tech, 2202 Kraft Dr SW, Blacksburg, VA 24060, USA; c Department of Human Development and Family Science, Virginia Tech, Wallace Hall, 366, 295W Campus Dr #0416, Blacksburg, VA 24061, USA; d Department of Business Information Technology, Virginia Tech, Pamplin Hall, 880W Campus Dr Suite 1007, Blacksburg, VA 24061, USA; e Management Information Systems Department, San Diego State University, 5500 Campanile Dr, San Diego, CA 92182, USA; f Department of Management Information Systems, Thammasat University, 2 Phra Chan Alley, Khwaeng Phra Borom Maha Ratchawang, Khet Phra Nakhon, Bangkok 10200, Thailand; g Department of Management, Radford University, P.O. Box 6954, Radford, VA 24142, USA; h Department of Information Systems and Business Analytics, Loyola Marymount University, 1 Loyola Marymount University Dr, Los Angeles, CA 90045, USA; i Bloomberg School of Public Health, Johns Hopkins University, 615N Wolfe St, Baltimore, MD 21205, USA

**Keywords:** Safety concerns, Health informatics, Older adults, Injury preventions

## Abstract

Older adults are among the fastest-growing demographic groups in the United States, increasing by over a third this past decade. Consequently, the older adult consumer product market has quickly become a multi-billion-dollar industry in which millions of products are sold every year. However, the rapidly growing market raises the potential for an increasing number of product safety concerns and consumer product-related injuries among older adults. Recent manufacturer and consumer injury prevention efforts have begun to turn towards online reviews, as these provide valuable information from which actionable, timely intelligence can be derived and used to detect safety concerns and prevent injury. The presented dataset contains 1966 curated online product reviews from consumers, equally distributed between safety concerns and non-concerns, pertaining to product categories typically intended for older adults. Identified safety concerns were manually sub-coded across thirteen dimensions designed to capture relevant aspects of the consumer's experience with the purchased product, facilitate the safety concern identification and sub-classification process, and serve as a gold-standard, balanced dataset for text classifier learning.

## Specifications Table

**Table:** 

Subject	*Health Informatics and Safety Research*
Specific subject area	*Older Adult Safety and Product Risk Identification*
Type of data	Text, Table, Figure
How the data were acquired	Data was obtained by extracting publicly available Amazon.com reviews through the use of an automated script. The software artifact was designed to identify and extract product reviews pertaining to the ‘assistive-aid’ category, which encompasses all relevant mobility assistive devices.
Data format	Raw, Analyzed
Description of data collection	The script extracted a total of 633,141 reviews, distributed across 3260 unique products (Amazon Standard Identification Numbers [ASINs]), from assistive-aid product categories often used by older adults (itemized in Table A.1 in Appendix A).
Data source location	Publicly available Amazon.com consumer product reviews for the assistive-aid product category.
Data accessibility	Repository name: Mendeley Data identification number: https://data.mendeley.com/datasets/jrs4sfnwyz/2

## Value of the Data

• A dataset with this volume of narratives does not exist in the older adult injury-prevention community. Researchers, manufacturers, and regulators alike may utilize the sub-coded narratives to identify safety concerns, prevent older adult mobility-related injuries, and extract valuable context that helps better understand product-risk vs personal-risk among older adults.
• The dataset was intentionally balanced by product category (equal number of safety concerns and non-concerns for each product type) to facilitate machine learning classifier training and testing.
• Trained classifiers can be used to automate the review labeling (sub-coding) process and quickly determine individual product risk levels across large datasets of online consumer reviews of products for older adults.
• Regulating agency product-risk early warning systems may better benefit from the use of a dataset focused around injury prevention, such as the one presented, rather than conventional, historic hospital-incident-reporting datasets.

## 1 Data Description

Although formal medical narratives describing consumer-product-related hospitalizations of older adults are retrospectively captured in the United States’ National Electronic Injury Surveillance System (NEISS), informal consumer narratives highlighting prospective safety concerns for these products have not been systematically collected. The dataset we describe here aims to fill this void. The data presented contains 1966 sub-coded Amazon.com mobility-related product reviews, distributed across eight mobility-related product categories (seen in Table 1). Each product category was intentionally stratified and balanced (equal number of safety concerns and non-concerns), to facilitate the usage in training and testing of machine learning classifiers. Additionally, within the file, safety concerns and non-concerns are separated across two worksheets, ‘Concerns Dataset’ and ‘Non-Concerns Dataset’, respectively. Identified concerns were manually sub-coded and classified according to the categorical and binary dimensions presented in the following section.

**Table 1:** Product categories (Assistive aid products).

Mobility-related products	Description
Kneewalkers & Scooters	knee walker and (knee) scooters.
Rollators & Walkers	rollators/walkers/walker accessories.
Canes	canes and hiking sticks.
Crutches	crutches/hands free crutches/crutch accessories/ankle braces.
Ramps	Ramps.
Wheelchairs & Transport Chairs	wheelchairs/transport chairs.
Car Accessories	car assistive devices.
Gaits & Transfer Belts	gaits and transfer belts.

### 1.1 Hazard narrative sub-coding categories

Hazard narratives are expressed throughout a total of 43 descriptive features. The first eleven features correspond to general review information, such as title and date posted, while the remaining thirty-two contain relevant sub-coded information regarding product performance, consumer injuries, design guidance, and author type. These sub-coded features are split across a number of categorical (5) and binary dimensions (7), which are designed to capture relevant aspects of the customer's narrative with the purchased product (Fig. 2).

Categorical dimensions (described below) contain information concerning body part affected, injury type, severity, product performance, and design guidance:

○ *Injury timing:* Injury severity (none, potential, minor, major), as per definitions in Table B.1 in Appendix B.
○ *Injury type:* Code numbers, names, and definitions for the “Injury Type” dimension are from the CPSC NEISS Coding Manual, pg. 12 [2], and are replicated in Table B.2 in Appendix B for convenience.
○ *Body part affected:* Code numbers, names, and definitions for the “Body Part Affected” dimension are from the CPSC NEISS Coding Manual, pg. 16 [2], and are replicated in Table B.3 in Appendix B for convenience.
○ *Pathway to injury:* Product performance defect that led to injury, coded according to Table B.4 in Appendix B.
○ *Design guidance:* Customer suggested product alterations, coded according to Table B.5 in Appendix B.

Binary features document consumer product-related falls, guidance type, author type, and specific product defects, namely, pain while using (DSPI), part breakage, and poor surface handling:

○ *Safety concern:* Coded as 1 when Injury Timing and Severity dimension was Potential Injury, Minor Injury, or Major Injury; coded as 0 otherwise. That is, coded as 0 only when Injury Timing and Severity dimension was No Injury.
○ *Fall:* Coded as 1 when consumer described occurrence of a fall by themselves or a fall by someone using the product; coded as 0 otherwise. Example: “My 91-year-old father has had this item for less than 9 months and he fell when getting out of bed”.^1^
○ *Part Breakage*^2^**:** Coded as 1 if the review clearly states a piece broke off or fell off the product; coded as 0 otherwise. Examples: “The scooter scooted fine for 6 weeks. Then **the front wheel assembly snapped off**. The metal literally cracked and separated.”, “**Wheel fell off** after 2 months. Had to buy replacement bolts at ACE hardware to repair.”
○ *DSPI:* Coded as 1 when user experiences consistent Discomfort, Soreness, Pain, or Irritation while using the product; coded as 0 otherwise. Examples: “The lettering on the cushion **hurt my knee** to the point it made my knee bleed” or “Had to take it off my chair because it **scrapped my knuckles** every time I would push up out of my chair.
○ *Poor surface handling:* Coded as 1 if product handling performance is unsatisfactory on different surfaces, product keeps getting caught, or is a hazard on different surface types; coded as 0 otherwise. Examples: “if you go over a rumble strip or an incline **be prepared to fall**”, “quite effectively and painfully **delivers any shock from rough surfaces** right to the injured ankle! And their small narrow size **makes them easily ‘chock.’** A **lamp cord or piece of gravel can send you tumbling** if you are not careful!”
○ *Design guidance type:* Coded as 1 if the customer provided explicit design guidance; coded as 0 if the design guidance had to be inferred. Example of explicit design guidance: “**If the walker would have attached easily** I think we might have avoided this disaster.”
○ *Author type:* Stored as two separate binary columns, capturing whether the review author explicitly mentions they are an *older adult* (Example: “tripping hazard **for me (80+ years old)**”) or they are a *caregiver to an older adult* (Examples: “**My mother** should have had her walker …”, “**My husband** fell because …”.

**Fig. 1:**
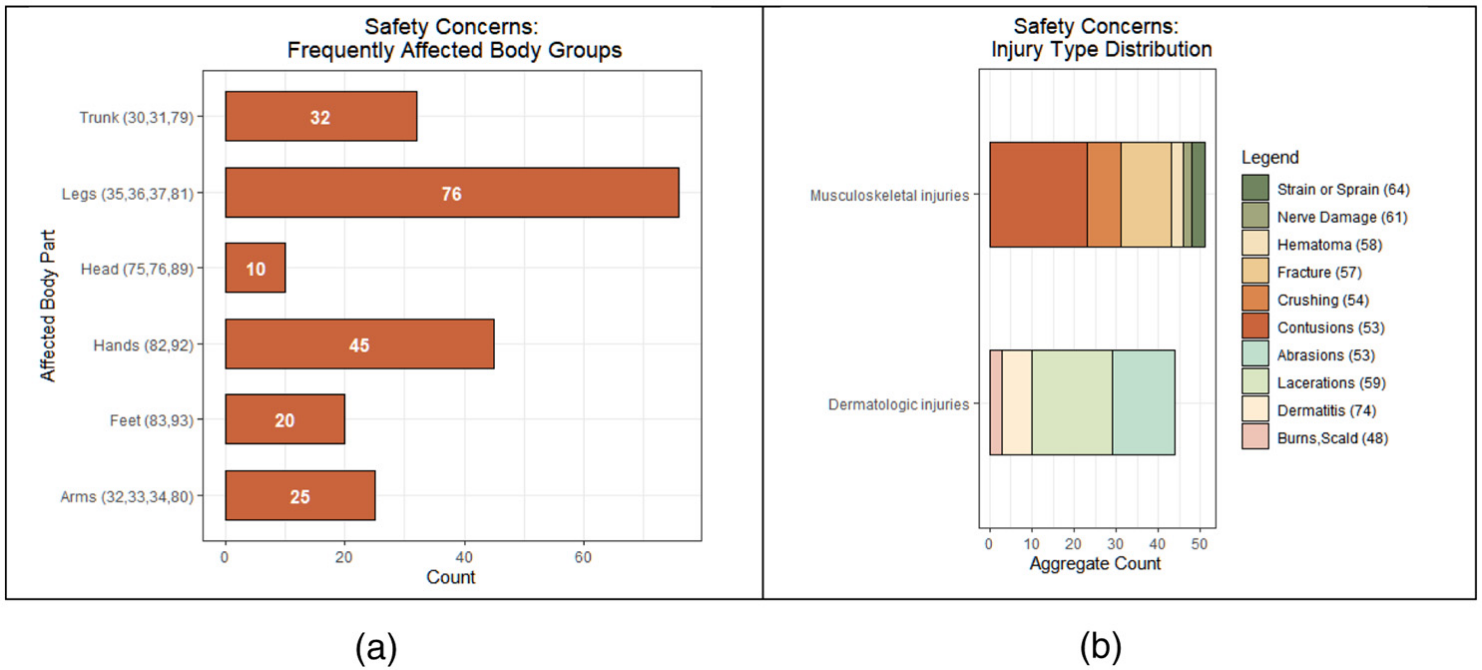
Frequently recorded affected body groups (a) and injury type (b) Distributions (count of reviews); non-reported body affected and non-stated injury type are excluded.

**Fig. 2:**
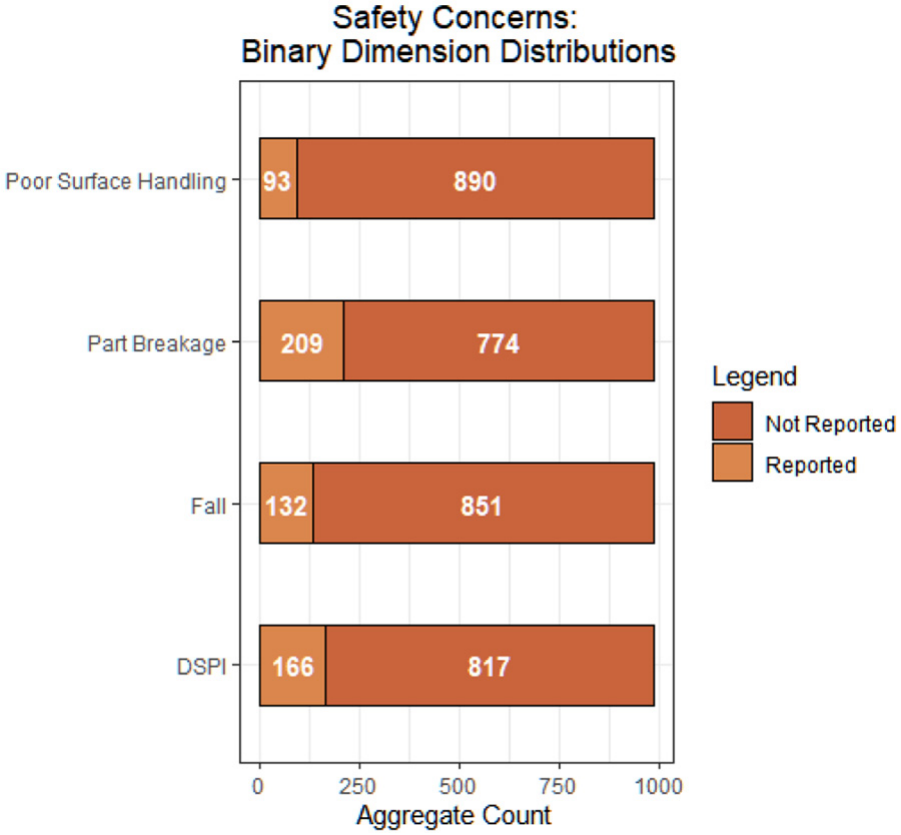
Safety concern binary dimension Distribution and aggregate counts (total reviews).

**Fig. 3:**
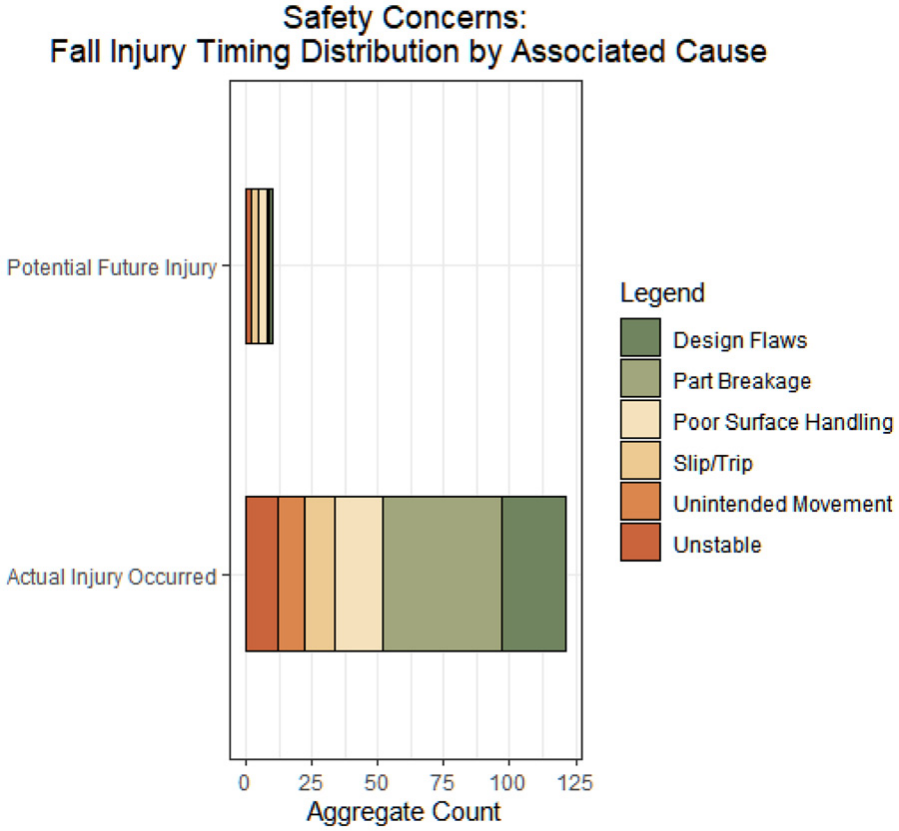
Product-related consumer fall breakdown by associated cause (count of reviews).

Table 2 shows five example safety-concern reviews from the final dataset, with the major coded attributes alongside, for illustration. Additionally, detailed dimension summary statistics can be found within the “Safety Concern Counts” sheet, available in the labeled (sub-coded) file, and in Fig. 1, Fig. 2, Fig. 3, and Tables 3 and 4.

**Table 2:** Labeled review samples, showing a selection of major coded attributes.

Review	Injury Timing	Injury Type	Body Part Affected	Pathway to Injury	Fall	Part Breakage	DSPI	Poor Surface Handling	Author Type: Caregiver
“...The next day at work, it collapsed and she broke her arm and hurt her other arm. We figured it may have just not locked right so we put it back together and made sure everything was right. This morning she went back to work and another piece fell off. This thing is junk.”	Major Injury	57 Fracture	33 Arm	3 Design, Material or Manufacturing Flaw	1	1	0	0	1
“Purchased for my elderly aunt and it is cheap and slipped out from under her. She fell and broke her hip and when we tried to return it we were told it was too late. That is because she was in the hospital for almost a month…”	Major Injury	57 Fracture	79 Trunk lower	2 Slip or Trip Hazard	1	0	0	0	1
“My mom's arms drag on the wheels when she uses the arm rests.”	Minor Injury	71 Other/Not Stated	33 Arm	3 Design, Material or Manufacturing Flaw	0	0	1	0	1
“…It sometimes gets off-balance, especially when turning. And the most annoying is my good right foot keeps catching on the back right wheel, cutting my right ankle bone…”	Minor Injury	59 Laceration	37 Ankle	3 Design, Material or Manufacturing Flaw, 4 Unstable	0	0	1	0	0
“This thing is a death trap. It does not come with instructions, the wheels are wobbly and uneven, there are no brake locks, if you go over a rumble strip or an incline be prepared to fall.”	Potential Future Injury	71 Other/Not Stated	87 Not recorded	1 Unintended Movement, 4 Unstable	0	0	0	1	0

**Table 3:** Pathway to injury and injury timing distribution (count of reviews).

Pathway to Injury	Actual Injury Occurred	Potential Future Injury
Unintended Movement	27	159
Slip or Trip Hazard	20	74
Design, Manufacturing, Material Flaws	241	506
Unstable	42	192

Total	330	931

**Table 4:** Product subcategory and injury timing distribution (count of reviews).

Product Category	Actual Injury Occurred	Potential Future Injury
Canes	43	224
Car Accessories	1	4
Crutches	83	82
Gaits & Transfer Belts	5	12
Ramps	2	10
Rollators & Walkers	52	213
Kneewalkers & Scooters	59	69
Wheelchairs & Transport Chairs	34	90

## 2 Experimental Design, Materials and Methods

Data was obtained by extracting Amazon.com reviews through the use of an automated script. The script extracted a total of 633,141 reviews, distributed across 3260 unique products (Amazon Standard Identification Numbers [ASINs]), from assistive-aid product categories often used by older adults (itemized in Table A.1 in Appendix A). In the Initial Exploratory Phase of data coding (described in detail under *Technical Validation: Initial Exploratory Phase* later on) 50,000 randomly selected reviews were labeled, in order to detect and flag potential safety concern narratives. A total of 3100 flagged narratives were shortlisted from the large-scale labeling effort.

### 2.1 Product category examination

Prior to beginning safety concern sub-coding, one of the investigators, a full professor in Human Development and internationally recognized expert in gerontology at an R1 University in the United States, examined the shortlisted review product categories. Examination efforts resulted in the identification of nineteen major product categories, seen in panel (a) of Table 5. Categories determined to be unrelated to mobility-assistance, such as shower aids – seen in panel (b) of Table 5 – were removed from the dataset, leaving 1045 potential safety concern narratives for mobility-related products for older adults. While most categories in panel (a) of Table 5 contain products predominantly used by older adults (e.g. rollators, walkers, canes, wheelchairs, and ramps), some categories (e.g. kneewalkers, scooters, and crutches) comprise products that are frequently also used by younger adults. The latter categories were also retained as there was occasional use by older adults and the safety-related consumer narratives can be regarded as helpful regardless of the age of the writer of the product review. The retention of solely mobility-related products allowed for the creation of finely tailored, category-specific sub-coding dimensions, facilitating the construction of a high-quality dataset.

**Table 5:** Shortlisted retained and removed product categories (assistive aid products).

(a) Categories Retained		(b) Categories Removed	
Mobility related categories	Description	Removed categories	Description
Kneewalkers & Scooters	kneewalkers and scooters	Toilet Aids & Commodes	raised seats/commodes/toilet stools/urinals/bedpans

Rollators & Walkers	rollators/walkers/ walker accessories	Shower Aids	grab, balance, safety bars/chairs/benches/ mats/hair washing basins

Canes	canes and hiking sticks	Bedroom & Daily Living	overbed tables/rails/ low strength assists/step stools

Crutches	crutches/hands free crutches/crutch accessories/ankle braces	Reaching Aids	grabbers/reach tools

Ramps	ramps	Pillows, Cushions, Wedges	cushions, wedges, mattresses, memory foam, pillows

Wheelchairs & Transport Chairs	wheelchairs/transport chairs	Nail Clippers & Hardeners	nail clippers & nail hardeners

Car Accessories	car assist devices	Magnifiers & Straws	magnifying devices and straws

Gaits & Transfer Belts	gaits and transfer belts	Hearing Aids	hearing aids/amplifiers/batteries

		Dressing Aids	shoehorns/stocking donners/dressing aids

		Misc	other/cast protectors/waterproof covers/writing aids/batteries/flashlights

### 2.2 Data coding (confirmatory phase)

In the Confirmatory Phase, two graduate assistants, at an R1 public land-grant university in the United States, individually labeled the 1045 shortlisted potential safety concerns, according to Injury Type (described in the previous section), confirming that 983 reviews were indeed safety concerns. The graduate assistants then further sub-coded features across a number of categorical and binary dimensions (described in the previous section). Categorical dimensions contain information concerning injury timing, body part affected, injury type, injury severity, design guidance, and general product performance attributes that relate to the safety concern (e.g. slippage and breakage). N-ary dimensions were reduced to multiple binary dimensions. For example, Body Part Affected is reduced into binary columns for body groups such as head, arms, legs, and so forth. Binary features also capture consumer-product-related falls, guidance type, author type, and specific product defects, such as pain while using, part breakage, and poor surface handling. Coding of the Injury Type and Body Part Affected dimensions by the two graduate assistants in the Confirmatory Phase was performed according to the Consumer Product Safety Commission (CPSC) National Electronic Injury Surveillance System (NEISS) coding manual [2].

### 2.3 Technical validation: initial exploratory phase (50,000 reviews)

In the Initial Exploratory Phase, *safety-concern discovery volume* was prioritized. Coding was completed by two hundred and twenty-nine (229) graduate (Masters-level) students at an R1 public land-grant university in the United States. We assessed inter-rater reliability via quadratic weighted Cohen's κ (1960) [1], as our labeling structure is best represented as that of an ordinal problem with varying degrees of disagreement between coders. For example, coding a review as “minor injury” versus “major injury” means the two coders were close and not totally discordant, whereas coding a review as “major injury” versus “no injury occurred” is a more significant disagreement. Hence, traditional Cohen's κ scores may not accurately reflect our inter-rater reliability, as they fail to factor in varying degrees of disagreement between coders.

Quadratic weighted scoring, popular because of its practical interpretations, assigns non-linearly distributed weights to ‘observed’ and ‘by chance’ probabilities, allowing for the representation of varying levels of agreement between raters and therefore providing a more accurate reading of our inter-rater reliability [3].

**Fig. 4:**
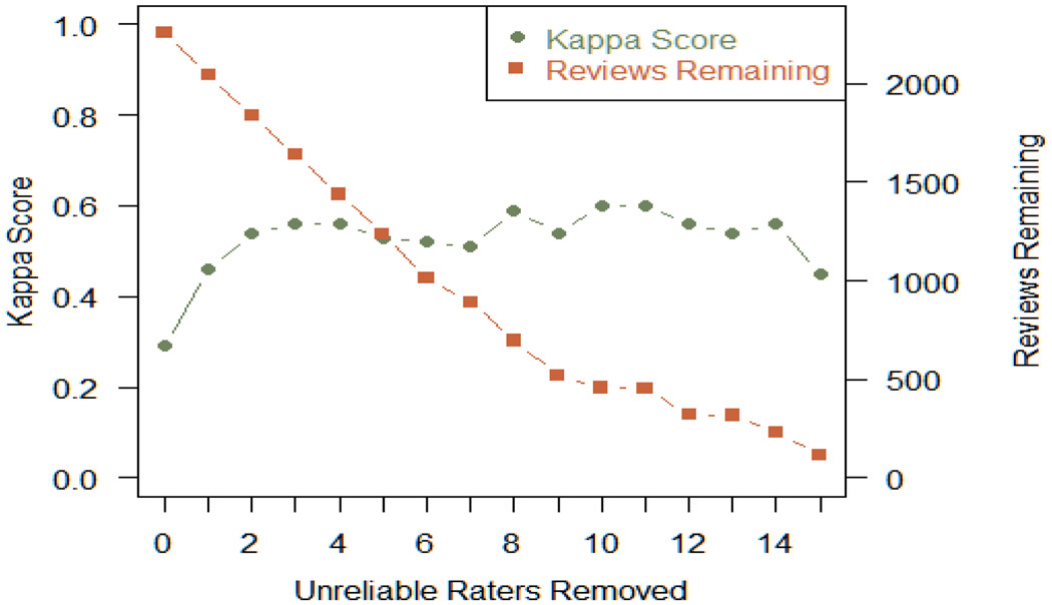
Kappa score and remaining reviews by unreliable raters removed.

Kappa scores were calculated by identifying double-coded reviews (2260) and filtering out unreliable coders who marked an unusually high proportion of reviews as safety concerns (“rogue coders”). This step generally accounts for most of the disagreements (see Fig. 4). Rogue coders were filtered out using the following criteria:

• More than 20% of their total labels are safety concerns.
• Tagged more than four reviews with ‘Major Injury’.

Filtering efforts resulted in the retention of 1258 double-tagged reviews, from which the weighted Cohen's κ scores were calculated. The weighted quadratic Kappa score for Injury Timing was κ = 0.59 (1258 cases; 133 disagreements; 1125 agreements; 89% agreement). Per Landis and Koch (1977) [4], these κ scores represent moderate inter-rater agreement, indicating satisfactory inter-rater reliability for the exploratory safety concern discovery phase. In the case of disagreement among coders, we allowed the most conservative decision to prevail; that is, an item was regarded as a safety concern if any coder regarded it as a safety concern.

Fig. 4, shown above, provides a visual representation of the effect unreliable coders have on Kappa scores, for the Initial Exploratory Phase coding. (For readability, the figure is truncated to reflect only the 15 most unreliable coders). The removal of two highly unreliable coders (x-axis) almost doubled quadratic Kappa scores (left y-axis), while still retaining just under 2000 double-tagged reviews (right y-axis), justifying rogue coder filtering efforts.

### 2.4 Technical validation: confirmatory phase (1045 safety concerns)

In the Confirmatory Phase, *safety-co*ncern labeling reliability was prioritized. The list of safety concerns shortlisted in the Initial Exploratory Phase was re-coded by two graduate assistants (Coder A and Coder B), and sub-coded along additional dimensions. Coder A and B first coded a random set of 72 shortlisted potential safety concerns (from the Initial Phase). For the initial 72 items, the Cohen's Kappa scores were κ = 0.68, 0.37, and 0.79 for the body part affected, injury type and injury timing dimensions, respectively. Both coders resolved discrepancies in their coding by discussion. A second random set of 72 items was coded to guarantee satisfactory Cohen's Kappa scores prior to final dataset labeling. The Cohen's Kappa scores were κ = 0.84, 1.00, and 0.61 for the body affected, injury type and injury timing dimensions, respectively. Once again, coders resolved discrepancies by discussion and then proceeded to continue coding the complete dataset in parallel. The Cohen's Kappa scores for the final dataset were κ = 0.95, 0.91, and 0.89 for the body affected, injury type and injury timing dimensions, respectively. Hence, there was further improvement in inter-rater agreement.

Table 6 shows the inter-rater agreement between graduate Coder A and graduate Coder B on the full set of 1045 items in the Confirmatory Phase.

**Table 6:** Cohen's Kappa scores for confirmatory phase.

Dimensions	Cohen's Kappa
Hazard Code - Body Affected	0.95
Hazard Code - Injury Type	0.92
Injury Timing	0.90
Fall	0.84
Pathway to Injury	0.87
DSPI	0.95
Surface Handling Issues	0.83
Design Guidance Type (Explicit vs Implicit)	.90
Design Guidance	.85
Author Type – Older Adult	1
Author Type - Caregiver	.90

Both coders discussed labels to resolve the remaining disagreements to arrive at the final labels for each record

## Ethics Statements

No conflict of interest exists in this submission. The authors declare that the work described in this paper is original and not under consideration for publication elsewhere. Its publication is approved by all the authors listed.

## CRediT authorship contribution statement

**Felipe Restrepo:** Writing – original draft, Data curation. **Namrata Mali:** Software, Validation, Writing – original draft, Data curation. **Laura P. Sands:** Conceptualization, Supervision, Writing – original draft, Formal analysis. **Alan Abrahams:** Conceptualization, Methodology, Supervision, Writing – review & editing. **David M Goldberg:** Software, Supervision, Project administration, Visualization. **Janay White:** Software, Formal analysis, Investigation, Supervision. **Laura Prieto:** Software, Formal analysis, Investigation, Supervision. **Peter Ractham:** Supervision, Project administration, Visualization. **Richard Gruss:** Supervision, Project administration, Visualization. **Nohel Zaman:** Supervision, Project administration, Visualization. **Johnathon P. Ehsani:** Supervision, Project administration.

## Declaration of Competing Interest

The authors declare that they have no known competing financial interests or personal relationships that could have appeared to influence the work reported in this paper.

## Figures and Tables

**Table A.1:** List of assistive-aid product sub-categories from Amazon.com. All categories fall under “Health & Personal Care, Medical Supplies & Equipment.”.

Category	Sub-Category	Sub-Category
Bedroom Aids & Accessories	Bed Safety Handles & Rails	
	Threshold & Wheelchair Ramps	
Mobility Aids & Equipment	Cane Accessories	Cane Flashlights
		Cane Holders
		Cane Ice Attachments
		Cane Tips
		Cane Wrist Straps
	Crutch Accessories	Crutch Handgrips
		Crutch Pads
		Crutch Tips
		General
	Mobility Scooters	
	Motorized Scooter Accessories	
	Rollators & Walkers	
	Walker Accessories	Baskets, Organizers & Pouches
		Replacement Wheels
		Ski Glides & Pads
		Trays
		General
	Wheelchair Accessories	Bags & Baskets
		Cushions
		Cushions
		Lap Trays
		General
	Wheelchairs	Electric Wheelchairs
		Self-Propelled Wheelchairs
	Mobility & Daily Living Aids	

**Table B.1:** Codes and definitions for “injury timing” dimension.

Injury Timing and Severity	Definition
No Injury	There is no indication of an actual or potential injury as a result of product usage. Example: “A real lifesaver after Achilles Tendon surgery. So happy with the product. It is sturdy and had no problems with corners or tipping.”
Potential Future Injury	Injury could possibly occur; thus, reviewer is cautious about using the product. Example: “I bought the bed cane for my mother who has Parkinson's. We thought it was awesome until **she nearly hung herself on it**. She slid off the bed and got her head caught under the hooked side. Luckily, I heard her before she suffocated.”
Minor Injury	Someone was actually hurt by the product, but it was a minor incident and no doctor's visit, or hospital visit was required. Example: “My 91-year-old father has had this item for less than 9 months and **he fell when getting out of bed**, after 2 pieces of it came off.”
Major Injury	Someone was actually seriously hurt by the product, and required a doctor or hospital visit, or died. Example: “The chair was assembled exactly as required in the instructions but was very uneven. My disabled husband used it and fell off requiring fire rescue and a **three day hospital stay**.”

**Table B.2:** Codes and definitions for “injury type” dimension.^3^

Injury Type Hazard Code	Definition
50 - Amputation	Amputation or risk of are identified.
48 - Burns, scald (from hot liquids or steam)	The specified burn type or risk of are identified.
51 - Burns, thermal (from flames or hot surface)	
52 - Concussions	Concussion or risk of are identified.
53 - Contusions, Abrasions	Bruises, scrapes, grazes or risk of either are identified.
54 – Crushing	Crushing (defined as severe external pressure), external pressure, or risk of either are identified. External pressure is limited to cases where the review explicitly states it was or could be the cause of injury/pain.
74 - Dermatitis, Conjunctivitis	The review associates the product with the onset of dermatitis or conjunctivitis. Dermatitis includes skin rashes, irritation, itchiness.
57 - Fracture	Fracture or risk of fracture are identified.
58 – Hematoma	Hematoma (a severe bruise where blood starts collecting or pooling under the skin) or risk of are identified.
59 - Laceration	Cuts, lacerations (defined as a severe cut), or risk of are identified.
61 - Nerve damage	Nerve damage or risk of are identified. Example: causing or aggravating carpal tunnel syndrome, sciatica pain.
68 – Poisoning	Poisoning or risk of are identified.
63 – Puncture	Puncture wounds, defined as wounds caused by sharp, pointed objects (nails, protruding screws) or risk of are identified.
64 – Strain or Sprain	Strain (tendon is overstretched or torn), sprain (ligament is overstretched or torn), or risk of either are identified.
71- Other/Not Stated	The injury type is not specified.

**Table B.3:** Codes and definitions for “body part affected” dimension.^4^

Body Group	Body Part Affected Hazard Code	Definition
Head (Neck Up)	75 - Head	Head injury or risk of are identified.
	76 - Face (including eyelid, eye area and nose)	Face injury or risk of are identified within the review, this includes eyelids, eyebrows, and the area immediately around the eyes.
	89 - Neck	A neck injury or risk of are identified.

Upper Trunk (Above Navel to Shoulders)	30 - Shoulder (including clavicle, collarbone)	A shoulder, clavicle, collarbone injury, or risk of either are identified.
	31 - Trunk, upper (not including shoulders)	An upper trunk, unspecified back injury, or risk of either are identified.

Arms (Wrist Included)	80 - Arm, upper	An upper arm injury or risk of are identified.
	32 - Elbow	An elbow injury or risk of are identified.
	33 - Arm, lower (not including elbow or wrist)	A lower arm, unspecified arm injury, or risk of either are identified.
	34 - Wrist	A wrist injury or risk of are identified.

Hands	82 - Hand	A hand injury or risk of are identified.
	92 - Finger	A finger injury or risk of are identified.

Lower Trunk (Below Navel to Pubic Region)	79 - Trunk, lower	A lower trunk, lower back (below the navel) injury, or risk of either are identified.
	38 - Pubic region	A pubic region injury or risk of are identified.

Legs (Ankle Included)	81 - Leg, upper	An upper leg injury or risk of are identified.
	35 - Knee	A knee injury or risk of are identified.
	36 - Leg, lower (not including knee or ankle)	A lower leg, unspecified leg injury, or risk of either are identified.
	37 - Ankle	An ankle injury or risk of are identified.

Feet	83 - Foot	A foot injury or risk of are identified.
	93 - Toe	A toe injury or risk of are identified.

Other	84 - 25–50% of body	25 – 50% of the body is identified as injured.
	87 - Not recorded	The review does not identify an affected body part.

**Table B.4:** Codes, definitions, and examples for “pathway to injury” dimension.

Pathway to Injury	Definition
1 - Unintended Movement	Brake related issues (failure, damage, lack of brake locks), canes/crutches falling by themselves, product moving when it was not supposed to. Example: ``The back seat locks do not work...**the back gives way when the person seated leans back** , a safety issue”

2- Slip or Trip Hazard	Product causes slipping/tripping, resulting in a potential fall hazard. Example: ``**hinges protrude above the surface of the ramp. This is definitely a tripping hazard**; I know because I caught my foot on it and fell''

3 – Design, Material, or Manufacturing Flaw	Product or product parts breaking/bending, poor material finishing (rough surfaces, sharp edges), pieces coming loose or falling off. General design issues such as product or product parts bumping into user, poor maneuverability (turning radius), inadequate height or width adjustments, missing pieces. Example: “the most annoying is **my good right foot keeps catching on the back right wheel**, cutting my right ankle bone” Example: “**Back wheel fell off.** When I went to attach the wheel that is when I **noticed the plastic groves were shredded.”** Example: “adjustable pin in the front that prevents the range of motion in the front axle. **It frequently works itself loose** allowing the pin to drop and letting the axel turn completely sideways.”

4 - Unstable	Product or product parts (directly relating to product stability, such as walker wheels) are unsteady, wobbly, tip over, or uneven. Example: “This thing is a death trap. It does not come with instructions; **the wheels are wobbly and uneven**” Example: "**Wobbly and unsafe**”

**Table B.5:** Codes and definitions for “design guidance” dimension.

Design Guidance (Improvement Type)	Definition
1 – Improve Design	Suggestions for the improvement of product design, e.g., wider base, leg length, weight.
2 - Improve Construction Quality	Suggestions for manufacturer, product assembly process improvement, e.g., wobbly/loose pieces, brake issues.
3 - Improve Material Quality	Suggestions for the use of better manufacturing materials, relates to product part breakage and overall product quality.
4 - Improve Comfort	Suggestions for the improvement of product comfort, e.g., increase product padding, surface softness.
5 - Add Non-Slip Surface	Suggestions for the addition of a non-slip surface, e.g., water-proof rubber tips, rubber wheels.
6 - Remove Sharp/Protruding Piece	Suggestions for the removal of protruding pieces, such as screws, or sharp edges from the product.
7 - Improve Surface Handling	Suggestions for the improvement of product performance on different surfaces.
8 – Other	Suggestions for other, customer-specified product improvements
9 – Implied Design Change Not Clear	Suggestions for product design change could not be determined from the provided information.

## Data Availability

A dataset of safety concerns narratives for injury prevention from online reviews of mobility-related products for older adults (original data) (Mendeley Data).

## Appendix B

Detailed sub-coding protocols followed by the graduate assistants throughout the Confirmatory Phase labeling process. Code numbers, names, and definitions for the “Body Part Affected” (Table B.2) and “Injury Type” (Table B.3, Table B.4, Table B.5) dimensions are from the CPSC NEISS Coding Manua [2], and are replicated here for convenience.

## Footnotes

3 The following CPSC NEISS codes were coded for, but have been omitted here for brevity, as zero occurrences of these codes were observed: 65 – Anoxia; 42 – Aspirated Foreign Object; 72 – Avulsion; 49 – Burns, Chemical; 73 – Burns, Radiation; 46 – Burns, Electrical; 47 – Burns, Not Specified; 60 – Dental Injury; 55 – Dislocation; 67 – Electric Shock; 56 – Foreign Body; 66 – Hemorrhage; 41 – Ingested Foreign Object; 62 – Internal Organ Injury; 69 – Submersion.

4 The following CPSC NEISS codes were coded for, but have been omitted here for brevity, as zero occurrences of these codes were observed: 94 – Ear; 77 – Eyeball; 88 – Mouth; 00 – Internal; 85 – All Parts of Body.

## Footnotes

1 In all examples, the bold emphasis is not in the original product review, but has been added here to highlight the phrase that is indicative of the relevant code.

2 While binary dimensions such as Part Breakage and DSPI are included within ‘3 - Design, Material, and Manufacturing Flaws’ under the “Pathway to Injury” dimension, their relatively high incidence rate and importance within the consumer's narratives warranted that they be separately documented in their own binary dimensions as well.
